# Epidemiology of Surgically Managed Mandibular Condylar Fractures at a Tertiary Referral Hospital in Urban Southwest China

**DOI:** 10.2174/1874210601711010294

**Published:** 2017-06-30

**Authors:** Swosti Thapa, Jun Wang, Hong-Tao Hu, Fu-Gui Zhang, Ping Ji

**Affiliations:** Department of Oral and Maxillofacial Surgery, Chongqing Medical University, Chongqing 401147, China

**Keywords:** Bone fractures, Condyle, Epidemiology, Mandible, Maxillofacial surgery, Trauma

## Abstract

**Background:**

Mandibular condylar fracture is one of the commonest maxillofacial fractures treated by maxillofacial surgeons. Demography of the patients, causation, and characteristics of the fracture depends on various socio-economic factors. Hence, maxillofacial surgeons should be familiar with epidemiology of mandibular condylar fracture.

**Objective:**

This study retrospectively describes the demography, etiology, fracture characteristics, and hospital utilization of surgically treated mandibular condylar fractures in a tertiary referral hospital in urban China in past five years.

**Methods:**

Data of all patients who underwent surgical management between 2011 and 2015 were collected. This included aetiology, characteristics of fracture, time, age, sex, associated injuries, and hospital utilization of 166 patients with 208 mandibular condylar fractures. These patients had undergone open reduction and internal fixation with either miniplates or lag screws. Among the fracture of head of mandibular condyle, 21.28% of the patients had the fracture segments removed. These data were statistically analyzed to describe the epidemiology of mandibular condylar fracture.

**Results:**

Most of the patients had unilateral mandibular condylar fractures (74.7%). Male patients (76.51%) outnumbered female patients (23.49%) in this cohort. The average age of the patients was 37 years. The fractures were mostly caused by fall from height (60.84%) and were located at the condylar neck (53.61%). Most of the patients had other associated maxillofacial injuries (71.08%) which were mostly located at symphysis and parasymphysis (44.59%). It took 12.58 +/- 0.35 days of hospitalization for the treatment.

**Conclusion:**

Fall from height was the most prevalent cause of mandibular condylar injury in mountainous urban China. The people at highest risk were middle-aged men. Mandibular condylar fracture was mostly located at the condylar neck and was usually associated with fracture at the symphysis and parasymphysis.

## INTRODUCTION

Mandible fractures are the commonest maxillofacial injuries with its epidemiology varying from country to country [1-4]. Mandibular fractures commonly occur at the condylar process with incidences reported between 18.39% to 42% [1, 2, 5, 6]. A number of epidemiological studies of mandibular trauma have guided the maxillofacial surgeons in anticipating and diagnosing traumatic mandibular fractures, but only few of the studies have focused on the condylar region.

The causes of mandibular fractures have varied with time and place which have been attributed to age groups, culture, lifestyles, temporal factors, and socioeconomic status. Physical assaults [1, 5-7], road traffic accidents [1, 2, 6-8], falls [6, 7], and sports-related injuries [9] are the major causes stated. Characteristics and location of mandibular fractures are related to the mechanism of injury and other variables such as age and gender. Similar pattern is expected in mandibular condylar fractures. Descriptive studies are the first step to understand the impact of health problem in a population. It is, therefore, imperative to understand the distribution, causation, and characteristics of mandibular condylar fractures for effective preventive and treatment efforts. This retrospective study aims to discuss the demography, causation, fracture characteristics, and hospital utilization of mandibular condylar fractures which were surgically treated at the Affiliated Stomatology Hospital of Chongqing Medical University over a five-years period. Affiliated Hospital of Stomatology of Chongqing Medical University is a tertiary referral center dedicated to dental surgery in Chongqing where cases needing specialist care are referred to from periphery hospitals. Chongqing is one of the youngest and most populous cities of China which lies in the mountainous region and has experienced the fastest socio-economic growth.

## MATERIALS AND METHODS

This retrospective study included patients who underwent surgical treatment of mandibular condylar fracture between January 2011 and December 2015 in the Department of Oral and Maxillofacial Surgery at the Affiliated Hospital of Stomatology of Chongqing Medical University. The patient’s identity, gender, age, etiology, date of injury, date of hospital admission, treatment received, date of treatment, date of discharge, characteristics of the fractures, and the associated injuries were noted from clinical records.

The demographic data of age and gender were statistically analyzed to describe any significant correlation to other variables of causation, hospital utilization, or fracture characteristics. The etiology of the mandibular condylar fractures included road traffic accident, fall from height, fall at ground level, assault, and work-place injuries. The fractures were classified into three sub regions according to specific landmarks and reference lines as fracture of condylar head, fracture of condylar neck, and fracture of condylar base [10]. The associated maxillofacial injuries were noted.

Those who had fractures of condylar neck and condylar base were fixed with titanium miniplates. Open treatment of fracture of head of mandibular condyle is controversial; but indications and treatment methods have been suggested in a number of studies [11-14]. Fractures of head of the mandibular condyle were approached by preauricular surgical approach and received either lag screws (61.7%) or titanium miniplates (17.02%) for osteosynthesis. Eleven cases of mandibular condylar head (21.28%) had the fragment segment removed by open method as described in previous studies [12, 15, 16]. The hospital utilization of patients was assessed as the interval between trauma and admission, between admission and surgery, and length of hospital stay.

The descriptive statistical analysis was conducted and figures generated using LibreOffice Calc spreadsheets. To analyze the relationships between multiple groups the Freeman-Halton extension of Fisher’s Exact Test and Student’s T test or ANOVA were performed. All hypotheses were verified at significance level equal to 0.05.

Ethical approval was sought and obtained, prior to commencement of the study taking place, from the ethics committee at the Affiliated Hospital of Stomatology of Chongqing Medical University Institutional Review Board.

## RESULTS

**Fig. (1):**
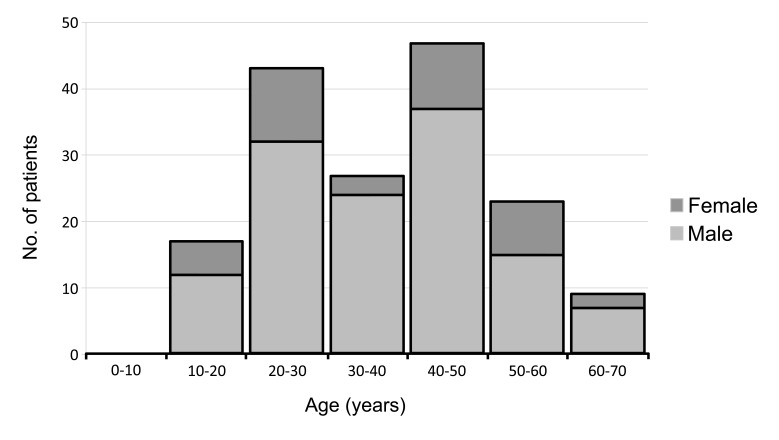
Patients’ age range from 12 to 70 years with most of the patients belonging to the age groups between 20 years and 50 years.

There were 166 patients who had presented with 208 mandibular condylar fractures that were operated in the study period between 2011 and 2015 at the Affiliate Hospital of Stomatology of Chongqing Medical University. One hundred and twenty four (74.7%) had unilateral and 42 (25.3%) had bilateral mandibular condylar fractures, and this ratio was not affected statistically by age, sex, nor causation of fracture. Male patients (76.51%) clearly outnumbered female patients (23.49%) (*p* <0.0001). The mean age of both the genders was identical (male *vs* female, 37.06 +/- 1.25 years *vs*. 37.13 +/- 2.43 years) with age ranging between 12 years and 70 years (Fig. **1**).

**Fig. (2):**
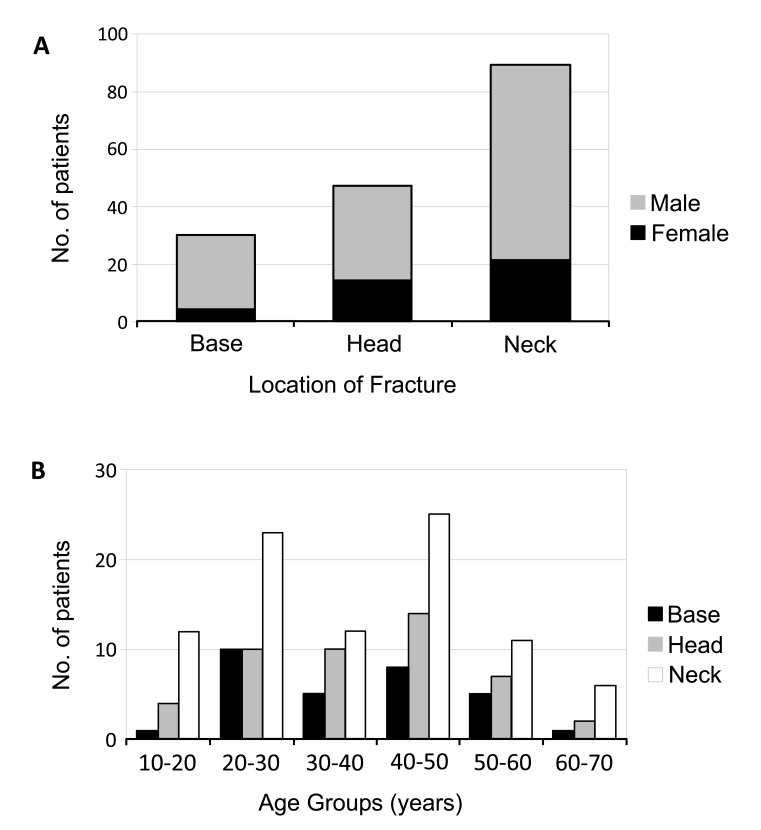
Location of fracture is classified into three subregions - fracture of the condylar base (18.07%), fracture of condylar head (28.32%), and fracture of condylar neck (53.61%). Fracture of neck was most prevalent in both the sexes (Fig. 2a) and in all age-groups (Fig. 2b).

Of the 166 patients, 47 (28.31%) had fracture of condylar head, 89 (53.61%) had fracture of condylar neck, and 30 (18.07%) had fracture of condylar base (Fig. **2a**). Fracture of condylar neck was seen more frequently in all age groups (Fig. **2b**) and both the sexes (Fig. **2a**).

**Fig. (3):**
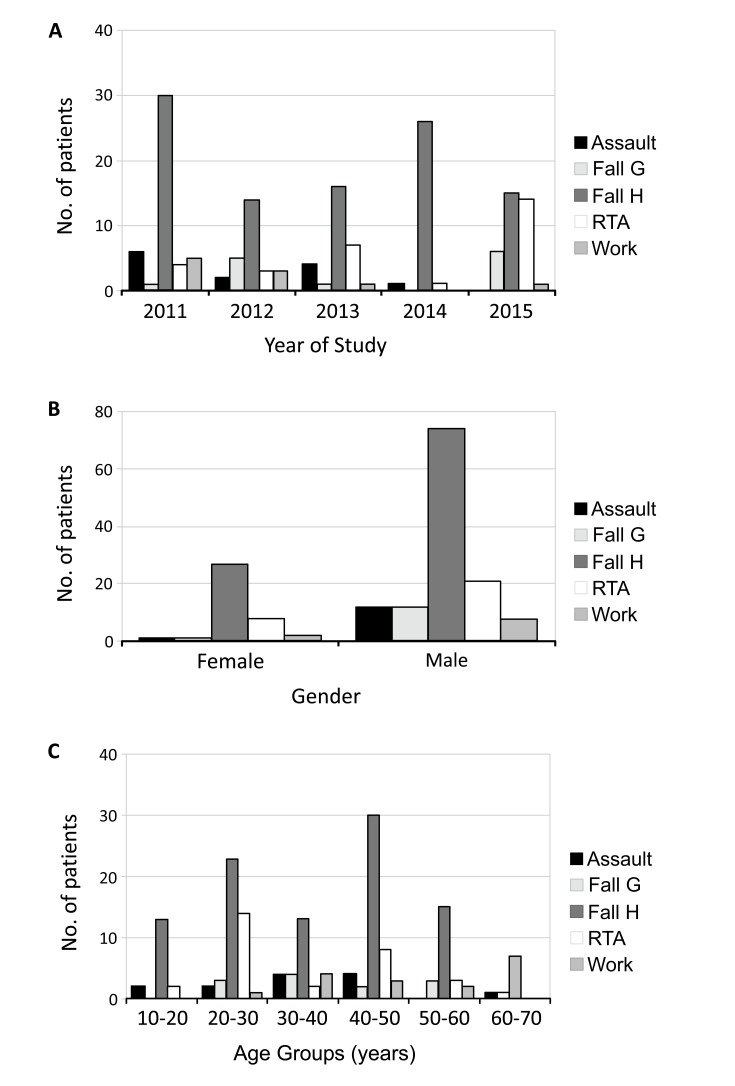
Assault, fall at ground level (Fall G), fall from height (Fall H), road traffic accidents (RTA), and work-related injuries caused the maxillofacial injuries. It was dominated by fall from height every year in the study period (Fig. 3a), in both the genders (Fig. 3b), and in all age-groups (Fig. 3c).

Fall from height (60.84%), road traffic accident (17.47%), assault (7.83%), fall at ground level (7.83%), and work-related injuries (6.02%) were the causes of maxillofacial trauma (Fig. **3**). Fall from height was the commonest cause of fractures in both male (58.27%) and female (69.23%) patients (Fig. **3b**) in all the age groups (Fig. **3c**). This was followed by road traffic accident which constituted only 17.47% of the total cases.

Forty eight (28.92%) condylar fractures were recorded without any other associated maxillofacial fractures. Seventy four (44.59%) condylar fractures were associated with fractures at mandibular symphysis and parasymphyseal area. Other maxillofacial injuries associated with mandibular condylar fracture were noted at mandibular body (12.65%), dentoalveolar region (8.43%), mandibular angle (7.23%), maxilla (3.01%), and zygomatic bone (4.22%) (Table **1**) .

**Fig. (4):**
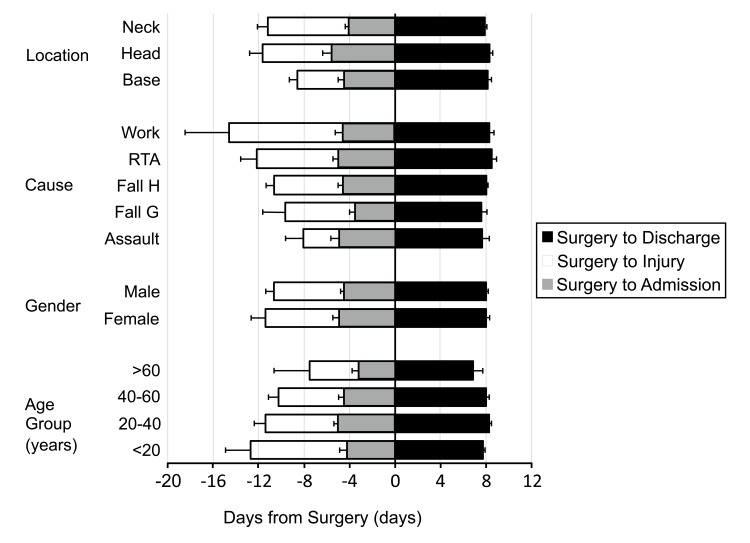
Mean days from injury to admission, admission to surgery and surgery to discharge were 6.24+/-0.61 days, 4.61+/-0.28 days and 7.98+/-0.17 days respectively with mean days of hospitalization totaling to 12.54+/-0.35 days. When compared between different categories, there were no statistically significant difference. (RTA, road traffic accident; Fall H, fall from height; Fall G, fall at ground level).

Of the 166 injured, 27 were admitted on the same day as the injury for the surgery; and 122 were admitted on the same week as the injury for the surgery. The mean number of days from injury to admission was 6.24+/-0.61 days. The patients admitted underwent surgery on 4.61 +/-0.28 days after the admission and were discharged 7.96+/-0.17 days after the surgery. This time periods did not significantly vary between age-groups, genders, fracture location, nor the cause of the fracture (Fig. **4**).

## DISCUSSION

Epidemiological studies of fractures guide in preventive, diagnostic, and therapeutic planning. This study was conducted in Chongqing which represents urban China. One hundred and sixty six patients who were admitted for surgical treatment for mandibular condylar fracture in a period of five years were analyzed. The male to female ratio in general population of Chongqing is 1.02 compared to 3.26 in our study population. Male predominance has been previously well established suggesting that men engage in activities of higher risk and suffer more severe mandibular trauma [1, 2, 6-8]. Mandibular condylar fractures were found to be more common in middle adulthood which could be assumed to be for the same reason.

Causes of mandibular injury who receive surgery vary in accordance with demographic and sociological factors and referral practices. Studies from different parts of the world have ranked falls [6, 7], road traffic accidents [1, 2, 6, 8], physical assaults [1, 5-7], and sports injuries [9] as major causes of the mandibular fractures. Falls contributed to three fifth of the mandibular condylar fractures (60.84%) in our study population with others contributing to only a small number. Fall from height has been identified as an important cause of trauma in mountainous Chongqing with most related to their work altitude and remaining related to daily life, suicide attempts, drug abuse, alcohol, and criminal behavior [17, 11]. Road traffic accident and physical assaults are also expected to be the major cause of fractures related to trauma in urban area. But, low incidence or road traffic accidents (17.47%) and assault (7.83%) in our population suggests efficiency of modern safety majors and prevailing law and order [8].

Fracture characteristics described depend on the mechanism and severity of the injury and the patient related variables. Symphysis and parasymphysis are the most common sites of injuries related to mandibular condylar fractures in different studies [7, 8]. Our study population suffered mandibular condylar fracture mostly due to falls with fractures located at the neck of the mandibular condyle and were most often related to fractures at the symphysis and parasymphysis. This was not statistically related to gender or the age of the patients.

Cost of the treatment and days of convalescence largely depend on the treatment received and hospital utilization. Treatment, to be effective, should be timely delivered at the most optimal cost and at the shortest possible days of hospitalization. These factors depend on individual institutional protocols that are tailored according to the pattern of injuries treated and treatment given. The parameters analyzed were days from injury to hospital admission and length of hospital stay which were found to be constant in the years studied and were affected by neither the patient nor the fracture characteristics.

Selection bias is a limitation of this study towards more severe injuries because the patients with mandibular condylar fractures who did not require surgical management were not represented in this study.

## CONCLUSION

This is a descriptive study of patients with mandibular condylar fracture who were treated with open surgical intervention at a single tertiary referral center in mountainous urban China. These fractures were mostly due to fall from height. The middle-aged men were found to be the ones at the highest risk of such injury. Most of the fractures occurred at the neck and were associated to injury at the symphysis and parasymphysis. Appropriate surgical treatment done as per the protocol followed at the institute required an average of 12 days of hospital stay.

## Figures and Tables

**Table 1:** No of cases and percentages of fractures of condylar base, fractures of condylar head, and fractures of condylar neck associated with fractures at other regions.

	Injuries Associated with Mandibular Condyle Fracture						
	None	Dentoalveolar	Mandibular Angle	Mandibular Body	Symphyseal/ Parasymphyseal	Maxilla	Zygomatic
Base	6 (20%)	3 (10%)	3 (10%)	2 (6.67%)	16 (53.33%)	0 (0%)	1 (3.33%)
Head	10 (21.28%)	4 (8.51%)	5 (10.64%)	5 (10.64%)	26 (55.32%)	0 (0%)	2 (4.25%)
Neck	32 (35.96%)	7 (7.87%)	4 (4.49%)	14 (15.73%)	32 (35.96%)	5 (5.62%)	4 (4.49%)
